# Measuring flushing symptoms with extended-release niacin using the flushing symptom questionnaire©: results from a randomised placebo-controlled clinical trial

**DOI:** 10.1111/j.1742-1241.2008.01739.x

**Published:** 2008-06

**Authors:** J F Paolini, Y B Mitchel, R Reyes, S Thompson-Bell, Q Yu, E Lai, D J Watson, J M Norquist, C McCrary Sisk, H E Bays

**Affiliations:** 1 Merck Research Laboratories Rahway, NJ, USA; 2 Merck Research Laboratories North Wales, PA, USA; 3 Louisville Metabolic and Atherosclerosis Research Center Louisville, KY, USA

## Abstract

**Introduction:**

Niacin is underutilised because of flushing. Lack of a quantitative tool to assess niacin-induced flushing has precluded the objective evaluation of flushing associated with extended-release (ER) niacin formulations. We developed the Flushing Symptom Questionnaire^©^ (FSQ), a quantitative tool to assess patient-reported flushing, and assessed its ability to characterise ER niacin-induced flushing.

**Methods:**

This study focused on the responses to one question in the FSQ, the Global Flushing Severity Score (GFSS), reported on a 0–10 scale (none = 0, mild = 1–3, moderate = 4–6, severe = 7–9 and extreme = 10) to assess flushing during ER niacin initiation (week 1) and maintenance (weeks 2–8).

**Results:**

Flushing severity with ER niacin was greatest during week 1 and remained greater than placebo for the study duration. During weeks 2–8, 40% of patients on ER niacin vs. 8% of those on placebo had > 1 day/week with ‘moderate or greater’ GFSS.

**Conclusions:**

In conclusion, the GFSS component of the FSQ was a sensitive and responsive quantitative measure of ER niacin-induced flushing that will aid in the objective comparison of novel strategies intended to improve tolerability and adherence to niacin, an agent proven to reduce cardiovascular risk.

**Disclosures:**

John F. Paolini, Yale B. Mitchel, Robert Reyes, Sally Thompson-Bell, Qinfen Yu, Eseng Lai, Douglas J. Watson, Josephine M. Norquist and Christine McCrary Sisk are employees of Merck & Co., Inc., and may own stock/hold stock options in the Company. Dr Harold Bays has served as a Clinical Investigator for (and has received research grants from) the following: Abbott, Alteon, Arena, AstraZeneca, Aventis, Bayer, Boehringer Ingelheim, Boehringer Mannheim, Bristol Myers Squibb, Ciba Geigy, Eli Lilly, Esperion, Fujisawa, GelTex, Genentech, GlaxoSmithKline, Hoechst Roussel, Hoffman LaRoche, InterMune, KOS, Kowa, Lederle, Marion Merrell Dow, Merck, Merck Schering Plough, Miles, Novartis, Parke Davis, Pfizer, Pliva, Purdue, Reliant, Roche, Rorer, Regeneron, Sandoz, Sankyo, Sanofi, Searle, Shionogi, Schering Plough, SmithKline Beacham, Takeda, TAP, UpJohn, Upsher Smith, Warner Lambert and Wyeth-Ayerst. He has also served as a consultant, speaker, and/or advisor to and for Arena, AstraZeneca, Aventis, Bayer, Bristol Myers Squibb, KOS, Merck, Merck Schering Plough, Metabasis Therapeutics, Microbia, Novartis, Nicox, Ortho-McNeil, Parke Davis, Pfizer, Roche, Sandoz, Sankyo, Sanofi Aventis, Shering Plough, SmithKline Beacham, Takeda, UpJohn and Warner Lambert.

**What's known**

Niacin has favourable effects on the lipid profile; however, its use in clinical practice has been hampered by bothersome side effects, primarily flushing. The lack of a quantitative tool to assess flushing symptoms has precluded the objective evaluation of niacin-induced flushing.

**What's new**

This study describes the utility of the Flushing Symptom Questionnaire^©^ (FSQ), a quantitative tool to assess patient-reported flushing end-points. The results show that the FSQ is a useful patient-reported outcome measure to objectively assess flushing associated with extended-release niacin.

## Introduction

Niacin (nicotinic acid), an agent with favourable effects on lipids, has been shown in clinical trials to reduce cardiovascular events or atherosclerosis progression in patients with cardiovascular disease and dyslipidaemia (1). The use of niacin in clinical practice has been hampered by poor tolerability, primarily because of flushing, which occurs to some degree in nearly all patients receiving niacin therapy (2–5). Although extended-release (ER) formulations of niacin reduce flushing symptoms relative to immediate-release formulations (6), the occurrence of flushing remains, requiring initiation at a low dose (i.e. 500 mg per day in the USA) with incremental increases to a therapeutically effective dose (2 g per day) after a period of months. Despite gradual titration, failure to achieve an optimal ER niacin dose and discontinuation of therapy frequently occur. Indeed, observational data show higher rates of discontinuation with ER niacin in clinical practice than those reported in clinical trials (7–9), signalling the need for improved formulations whose development would be enhanced by objective measures of ER niacin-induced flushing.

The Flushing Symptom Questionnaire^©^ (FSQ) is a quantitative tool to assess aspects of the severity, frequency, duration and bother of flushing (including symptoms of skin redness, warmth, tingling and/or itching separately and in aggregate). The FSQ was developed with input from clinical experts and through interviews with patients who had experienced flushing with current ER niacin-based products. It was pilot tested in patients for content and comprehension and validated using the data from this study, described elsewhere (10). The purpose of the present study was to evaluate the utility of the FSQ to characterise the severity of patient-reported ER niacin-induced flushing symptoms during initiation of ER niacin therapy and with persistent use.

## Methods

### Patient selection criteria

Male and female patients 18–70 years of age for whom ER niacin treatment was appropriate were eligible to be randomised. Other inclusion criteria were triglycerides (TG)≤ 27.5 mmol/l, alanine aminotransferase or aspartate aminotransferase (ALT or AST) < 1.5 × upper limit of normal (ULN), creatine phosphokinase (CK) < 2 × ULN, normal thyroid stimulating hormone as assessed by the investigator and based on the central laboratory reference range of 0.300–5.000 μIU/ml, haemoglobin A_1C_≤ 8.5%, and commitment to maintaining a hand-held electronic diary (e-diary). Non-study lipid-lowering medication was permitted provided that the dose was stable during the study. Low-dose aspirin (81 mg) dosed in the morning was allowed for cardiac prophylaxis.

Patients with confounders for flushing (e.g. postmenopausal women with a history of flushing, niacin use > 50 mg/day, or aspirin use > 81 mg) or prior history of hypersensitivity or intolerance to niacin were excluded from the study. Medications to ameliorate or prevent flushing symptoms were not permitted.

### Study design

**Figure 1:**
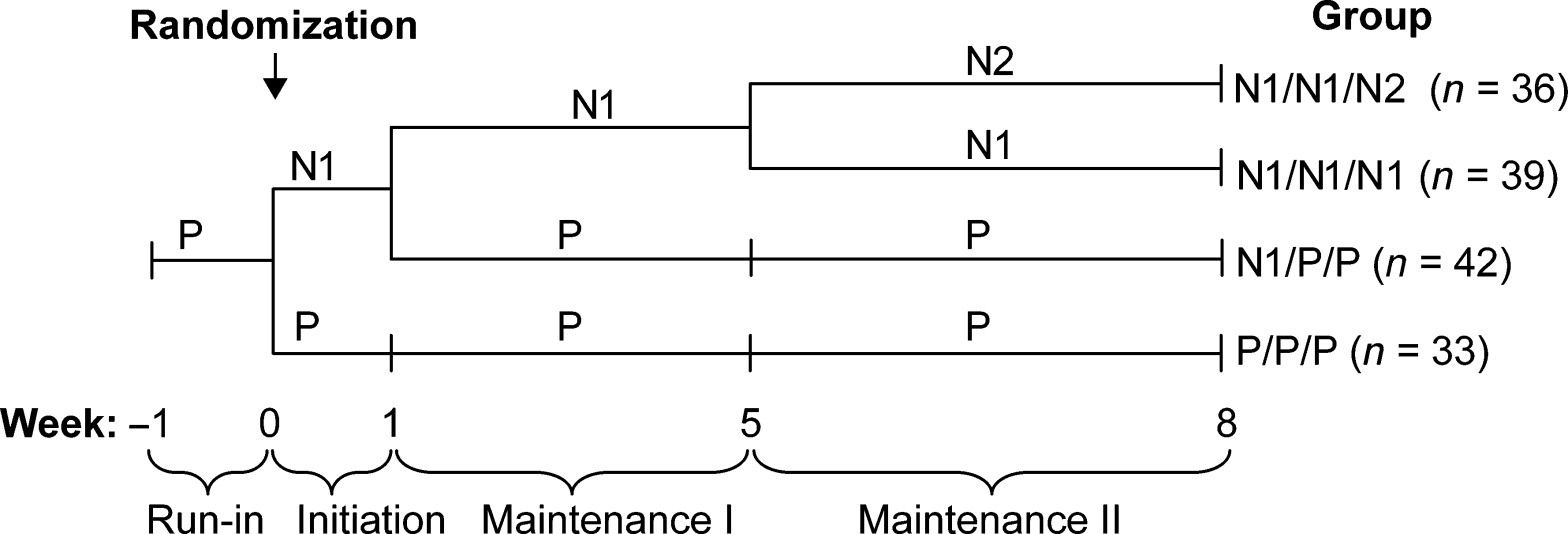
Study design. Patients were randomised to one of four treatment sequence groups, represented by the group designations shown at the end of each sequence. The group designations represent the treatment received during the Initiation, Maintenance I and Maintenance II Phases of the study respectively. N1 = extended-release niacin 1 g; N2 = extended-release niacin 2 g; P = placebo

This was an 8-week, multicentre, randomised, double-blind, placebo-controlled study to assess patient-reported ER niacin-induced flushing end-points during the initiation (week 1) and maintenance (weeks 2–8) phases of ER niacin (given as NIASPAN™; Abbott Laboratories, Abbott Park, IL) therapy. After completing a 1-week placebo run-in period, eligible patients were randomised in a 3 : 3 : 3 : 2 ratio to one of four treatment groups (Figure 1). Following randomisation, all groups underwent a 1-week initiation treatment phase of either ER niacin 1 g/day (i.e. without any titration dose; groups 1, 2 and 3) or placebo (group 4). Afterwards, group 1 was administered ER niacin 1 g for weeks 2–4 and then advanced to ER niacin 2 g (2 × 1 g ER niacin pills) for weeks 5–8 (N1/N1/N2). Group 2 was administered ER niacin 1 g for weeks 2–4 and then continued on ER niacin 1 g (+1 matching placebo tablet to maintain blinding) for weeks 5–8 (N1/N1/N1). Group 3 was switched to matching placebo for weeks 2–4, with a second placebo tablet to maintain blinding added for weeks 5–8 (N1/P/P). Group 4 received placebo matching ER niacin, one tablet for weeks 1–4 and two tablets for weeks 5–8 (P/P/P). All study medication was administered with the evening meal rather than at bedtime to allow adequate time for ER niacin-induced flushing symptoms to occur during the wakeful hours. A poststudy telephone visit was performed 14 days after discontinuation or completion visit of the study to assess for serious adverse experiences.

**Figure 2:**
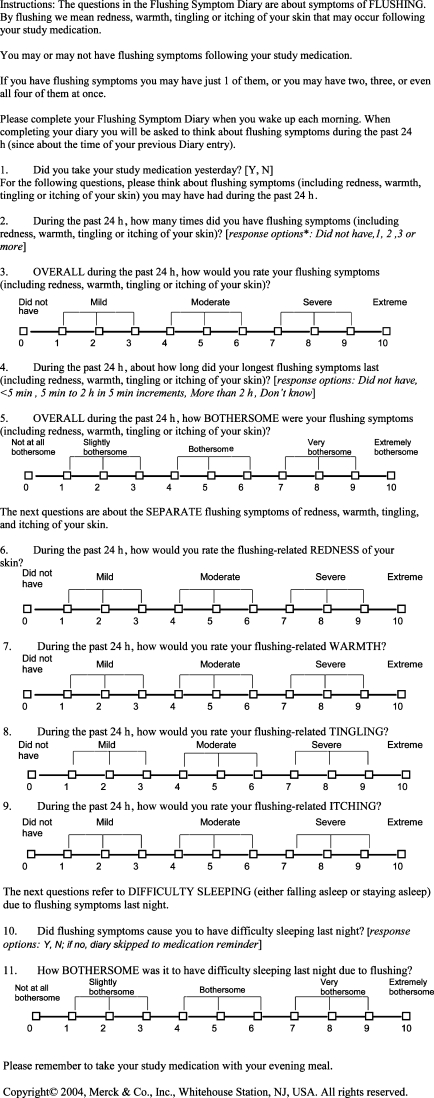
The Flushing Symptom Questionnaire^©^

The frequency, severity and bother of flushing (including symptoms of skin redness, warmth, tingling and/or itching), the duration of flushing and sleep disturbance because of flushing were assessed using the 11-question FSQ (Figure 2) provided via a hand-held e-diary. The e-diary was completed each morning, and the results were uploaded to a central database; the responses reflected the flushing symptoms (redness, warmth, itching and tingling) experienced during the previous 24 h. Item 3 of the FSQ, termed the Global Flushing Severity Score (GFSS), assessed flushing severity of all four symptoms in aggregate. In a separate FSQ validation analysis of data from the present study, the GFSS was selected as the best measure of flushing severity for use as an end-point in clinical trials of niacin-based therapy (10). This study reports the results of the GFSS to assess ER niacin-induced flushing in the initiation and chronic maintenance phases of treatment with ER niacin.

Measures to ensure compliance and maintain study retention included close patient follow-up (daily uploading of e-diary data, frequent clinic visits and drug accountability), educational materials informing patients about the characteristics of ER niacin-induced flushing, non-pharmacologic measures (that were allowed by the trial) to reduce the discomfort of ER niacin-induced flushing, and patient instruction regarding the importance of remaining in the trial through to completion to ensure an optimal assessment of flushing severity.

The study was conducted in accordance with the guidelines on good clinical practice and with ethical standards for human experimentation established by the Declaration of Helsinki. Ethics Review Committee/Institutional Review Board approval was obtained for each study site. Informed consent was obtained from all patients before any study procedure was performed.

### Study end-points

The primary end-points of the study included: (i) the maximum GFSS during the 1-week treatment initiation phase as a measure of flushing severity in the subjects receiving ER niacin 1 g during week 1 (allocation groups N1/P/P, N1/N1/N1 and N1/N1/N2 combined) compared with the placebo group and (ii) the percentage of days with a GFSS of moderate or greater (≥ 4) during weeks 2 through 8 as a measure of flushing severity during maintenance of therapy. Other study end-points included duration of flushing episodes and discontinuation because of flushing symptoms.

Safety assessments included the collection of adverse experiences (AEs), physical examination and vital signs. Laboratory safety measurements included blood chemistry (including AST, ALT and CK performed at all treatment visits), haematology and urinalysis.

Prespecified discontinuation criteria included: persistent ≥ 3× the ULN increases in ALT or AST; persistent five to 10-fold increases in CK with muscle symptoms; chronic (> 2 weeks) treatment with systemic corticosteroids; TG levels ≥ 55 mmol/l on repeat measure; positive pregnancy test; or any condition that exposed the patient to significant risk by continuing in the trial or did not permit the patient to adhere to the requirements of the protocol.

### Statistical methods

All randomised patients who took at least one dose of postrandomisation study drug were included in the analyses. The GFSS was calculated based on all available data within the respective time interval, and there was no imputation of missing data. Flushing in the initiation phase was assessed by the GFSS during week 1 as the maximum GFSS score (and separately average GFSS score) as a continuous variable and analysed using an ANOVA model with factor for treatment and the maximum GFSS categorised as none/mild, moderate, severe or extreme. Between-treatment comparison was made using the Cochran–Mantel–Haenszel test.

Flushing during various time intervals (e.g. weeks 2–8, weeks 2–4 and weeks 6–8) in the maintenance phase was assessed as the percentage of days with moderate or greater GFSS (GFSS ≥ 4) during the time interval as a continuous variable and analysed using an ANOVA model with factor for treatment and the average GFSS score (and separately maximum GFSS score) during the time interval and analysed using an ANOVA model with factor for treatment. For the weeks 2–4 time interval, the two treatment groups that received ER niacin 1 g during the first 4 weeks of treatment were pooled together; for time intervals beyond the first 4 weeks of treatment, each of the four treatment groups was analysed separately.

Duration of flushing episodes (in minutes) was derived as the median across each patient's daily report from all non-missing diary records within the time interval of interest. The percentages of patients who discontinued study medication because of cutaneous symptoms were summarised by treatment. Descriptive statistics, e.g. mean or median, including standard deviation, were provided for each treatment.

## Results

### Demographics and baseline characteristics

The demographics and baseline characteristics were generally similar across the treatment groups (Table 1). The randomised patient population had a mean age of 48.7 years, included more males than females (62% male vs. 38% female), was predominantly Caucasian (80.6%), and had a mean body mass index of 30.1 kg/m^2^.

**Table 1:** Demographics and baseline characteristics

Demographic/baseline parameter	Placebo (*n* = 33)	ER niacin 1 g → placebo (*n* = 51)	ER niacin 1 g (*n* = 45)	ER niacin 1 g → ER niacin 2 g (*n* = 46)	All patients (*n* = 175)
Mean age in years (SD)	48.4 (12.7)	46.1 (12.4)	51.1 (11.7)	49.7 (10.7)	48.7 (11.9)
**Gender**					
Number of women (%)	9 (27.3)	17 (33.3)	19 (42.2)	22 (47.8)	67 (38.3)
Number of men (%)	24 (72.7)	34 (66.7)	26 (57.8)	24 (52.2)	108 (61.7)
**Race**					
White (%)	25 (75.8)	41 (80.4)	34 (75.6)	41 (89.1)	141 (80.6)
Black (%)	3 (9.1)	3 (5.9)	4 (8.9)	0 (0)	10 (5.7)
Hispanic (%)	5 (15.2)	6 (11.8)	7 (15.6)	5 (10.9)	23 (13.1)
Other (%)	0 (0)	1 (2.0)	0 (0)	0 (0)	1 (0.6)
Mean BMI (kg/m^2^) (SD)	30.4 (5.8)	28.9 (5.1)	30.4 (6.3)	31.0 (7.7)	30.1 (6.3)

ER, extended-release; SD, standard deviation; BMI, body mass index.

### Initiation of treatment

**Figure 3:**
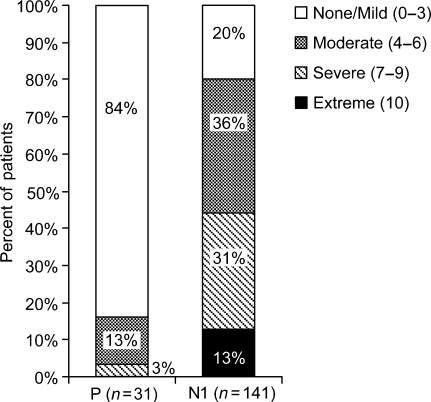
Maximum Global Flushing Severity Score in first study week (Initiation Phase). p < 0.001, based on Cochran–Mantel–Haenszel test

During week 1, patients receiving 1 g of ER niacin experienced significantly greater flushing compared with patients receiving placebo (p < 0.001 for between-group comparison for ER niacin 1 g vs. placebo; Table 2). The analysis of the maximum GFSS categorised during week 1 as none/mild, moderate, severe or extreme demonstrated statistically significant (p < 0.001) differences between the ER niacin 1 g and placebo groups (Figure 3).

**Table 2:** Maximum GFSS during week 1 by treatment group

		Maximum GFSS during week 1	Between-group difference relative to placebo in maximum GFSS during week 1	
Treatment group	*n*	LS mean (95% CI)	LS mean (95% CI)	p-value
P	31	1.1 (0.2, 2.1)		
N1	141	5.8 (5.4, 6.3)	4.7 (3.7, 5.8)	< 0.001

P, placebo; N1, extended-release niacin 1 g; GFSS, Global Flushing Severity Score; LS, least squares.

**Figure 4:**
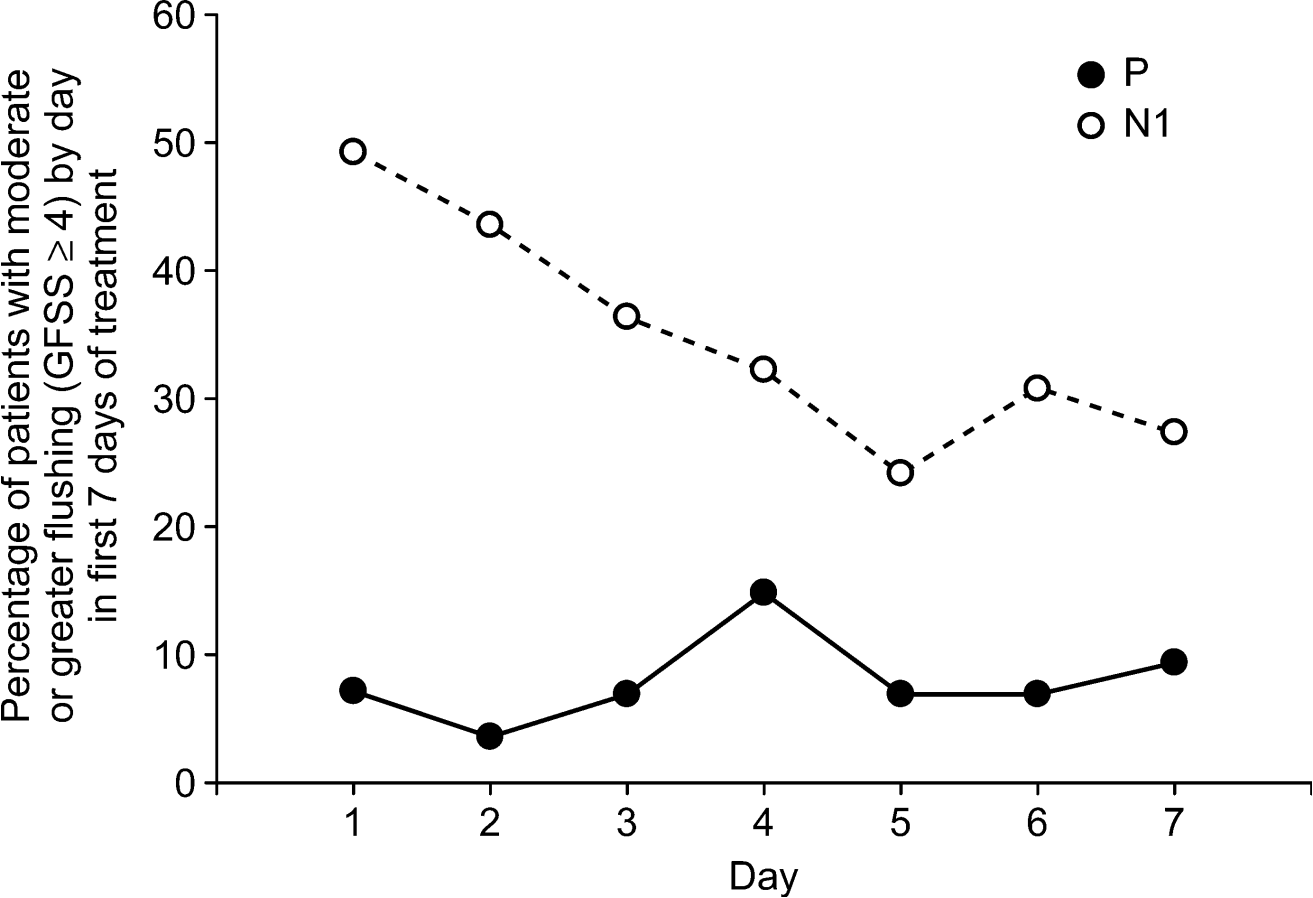
Percentage of patients with ‘moderate or greater’ flushing (Global Flushing Severity Score ≥ 4) by day in the first 7 days of treatment

The daily mean GFSS during the first 7 days of treatment was examined for the patients receiving ER niacin 1 g and those receiving placebo (Figure 4). Among patients receiving ER niacin 1 g, flushing severity was greatest during the first 2 days on treatment, with 49% and 43% of patients having GFSS ≥ 4 on days 1 and 2, respectively. Among patients receiving placebo, < 15% of patients had GFSS ≥ 4 on any of the first 7 days. The duration of flushing (minutes/day) analysis demonstrated that the median flushing duration was 0 min/day in the placebo group vs. 22.5 min/day in the ER niacin 1 g group.

### Maintenance treatment period

**Figure 5:**
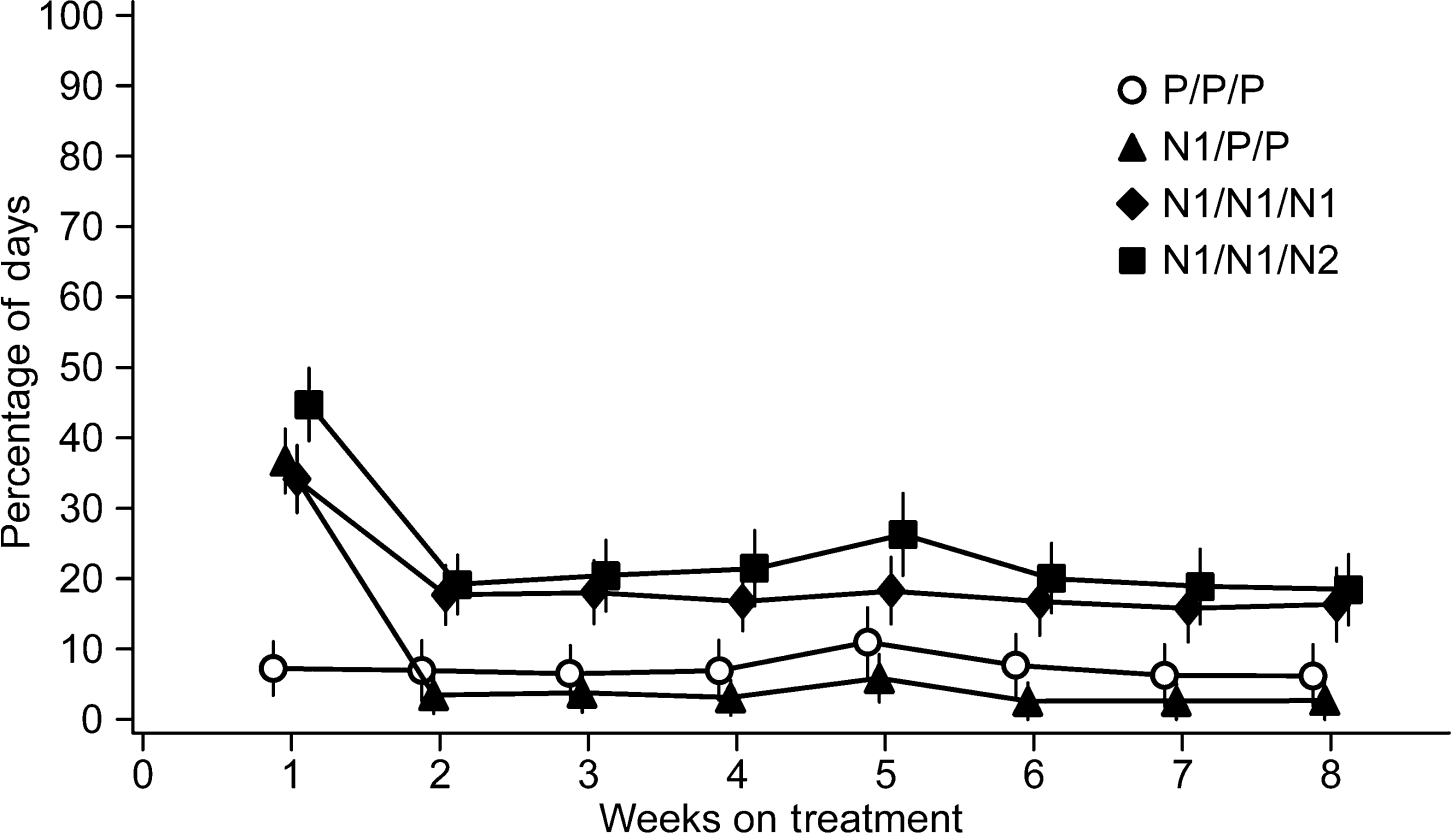
Percentage of days with Global Flushing Severity Score of moderate or greater, by treatment group and study week (mean ± standard error)

Patients who started on ER niacin 1 g in week 1 and who remained on ER niacin 1 g or 2 g thereafter reported the greatest flushing severity in the first week; their flushing severity decreased in the second week but remained relatively constant throughout the remaining weeks (Table 3, Figure 5). During weeks 2 through 8, approximately 40% (30 of 75) of the patients on ER niacin (1 g and 2 g combined) vs. 8% (six of 75) of the patients on placebo had more than 1 day per week with moderate or greater GFSS (Table 3). A small increase in flushing was observed when patients were advanced from 1 g to 2 g of ER niacin at week 5.

**Table 3:** Percentage of days with moderate or greater* GFSS during weeks 2 through 8

		Percentage of days with moderate or greater GFSS	Between-group difference relative to placebo	
Treatment group	*n*	LS mean (95% CI)	LS mean (95% CI)	p-value
P/P/P	33	7.2 (−0.4, 14.8)		
N1/P/P	42	3.3 (−3.4, 10.0)	−3.9 (−14.1, 6.2)	0.444
N1/N1/N1	39	17.5 (10.6, 24.5)	10.3 (0.05, 20.6)	0.049
N1/N1/N2	36	22.7 (15.4, 29.9)	15.5 (5.0, 25.9)	0.004

* Percentage of days during the specified study week(s) for which patients reported GFSS ≥ 4. P, placebo; N1, extended-release niacin 1 g; N2, extended-release niacin 2 g; GFSS, Global Flushing Severity Score; LS, least squares.

The weekly mean proportions of days with moderate or greater GFSS (≥ 4) were examined across treatment groups (Figure 5). In the N1/N1/N1 group, the mean weekly percentage of days with moderate or greater GFSS was 34.1% at week 1, and about 17.7% at week 2 and beyond. A similar profile was observed in the N1/N1/N2 group, with some increase in flushing at week 5 when the dose was advanced from 1 to 2 g.

The N1/P/P group had a higher percentage of days with moderate or greater GFSS during week 1; afterwards, the weekly percentages approximated those for the placebo arm. The placebo group had a low percentage of days with moderate or greater GFSS across the entire treatment period.

### Discontinuation because of flushing

Overall, 14/175 patients (8%) discontinued because of flushing, all of whom were exposed to ER niacin. Most (11/14) discontinuations because of flushing occurred during the first week of the treatment (initiation dose at 1 g). None of the patients on ER niacin 1 g with maximum GFSS during week 1 in categories none/mild or moderate discontinued the study because of flushing, while 15.9% and 22.5% of the patients with maximum GFSS during week 1 in categories severe and extreme, respectively, discontinued from the study within the first week of treatment because of flushing. After the first week, three more patients discontinued from the study because of flushing. Compliance with study drug (92%) and e-diary completion (87%) for the entire treatment period was high and not typical of ‘real-world’ experience with niacin therapy, presumably because this study was designed and monitored to maintain optimal compliance.

### Safety and tolerability

Overall, the safety profile was similar to that described in the NIASPAN label, despite that dosing was according to a new paradigm (1 g → 2 g over 5 weeks rather than a four-step, 500 mg increment titration to 2 g/day over 13 weeks). There were no drug-related serious AEs reported in this study and, with the exception of the observed flushing response that was recorded as an outcomes measure, ER niacin was well tolerated.

Two ER niacin-treated patients experienced ALT ≥ 2× but < 3× ULN (both in the 1 g group). Of the 175 patients who entered the active treatment period, only one had a laboratory AE (an increase in CK in the N1/P/P group). The elevation was determined by the investigator to be not drug related. There was no significant difference in the incidence of fasting blood glucose (FBG) values > 6.6 mmol/l (i.e. 20% greater than ULN) in the ER niacin-treated vs. placebo groups. There was a greater proportion of patients with FBG increases > 10% in the groups taking only ER niacin 1 g (51.5%) or 2 g (39.1%) than in the P/P/P group (30.3%) and N1/P/P groups (34.0%). There were no cases of new onset of type 2 diabetes reported by the investigator as an AE during the course of the trial. Greater proportions of patients had platelet count decreases > 10% with increasing exposure to ER niacin: such increases were observed in 18% of the N1/P/P group, 23% of the N1/N1/N1 group and 45% of the N1/N1/N2 group vs. 9% of the P/P/P group.

Flushing symptoms were collected as an outcomes measure when recorded by patients in the e-diary and as a safety end-point when defined by the investigator as a spontaneously occurring AE. Flushing was the most frequently reported drug-related AE, observed only in the groups treated with ER niacin. Although some patients in the placebo group reported flushing on their daily e-diaries, there were no investigator-defined AEs of flushing in the placebo group. The incidence of the drug-related AE of flushing increased with advancement of ER niacin dose.

## Discussion

In the present study, all of the predefined FSQ-based flushing end-points succeeded in differentiating ER niacin-treated from placebo-treated patients both during the initiation and maintenance phases of therapy. The usual ER niacin titration schedule was not employed; rather, a provocative ER niacin dosing regimen was selected to elicit flushing symptoms; consequently, the results of this study with the 1 g, 2 g ER niacin dosing regimen are not intended to be generalisable for informing the tolerability of branded ER niacin according to the approved product label (e.g. titration schedule consisting of 500 mg increases per 4-week period) (6). However, the FSQ adds value to the evaluation of the magnitude of the ER niacin-induced flushing signal from any niacin formulation, regardless of manner of dosing.

The primary comparison in the initiation phase (week 1) was the maximum flushing severity with ER niacin vs. placebo. Flushing categorised as moderate or greater was reported by 80% of patients taking ER niacin vs. 16% of patients taking placebo during this week. The proportion of patients treated with ER niacin who reported flushing in this study was consistent with that reported in other studies of ER niacin. Among patients who received ER niacin, the flushing severity over week 1 was highest during the first 2 days on treatment. In contrast, fewer than 25% of patients who received placebo during week 1 had any flushing (GFSS ≥ 1).

The observed placebo flushing response demonstrates that effective blinding controls were in place (i.e. patients were not able to identify that they were in the placebo group). Indeed, an ER niacin run-in period prior to separation of groups was employed to mitigate the risk of confounding the analysis in the maintenance phase by unblinding of placebo patients during the initiation phase. Moreover, the symptoms of flushing are commonly experienced for many reasons in addition to treatment with niacin, including the placebo effect of participating in an ER niacin-induced flushing study, underscoring the importance of a validated tool to objectively assess flushing. Nevertheless, patients in the placebo group experienced a shorter median duration of flushing than those in the ER niacin group (median duration 0 min/day vs. 22.5 min/day, respectively), suggesting that while the GFSS is the best measure of flushing severity, other questions in the FSQ are useful for discriminating the quality of flushing depending on aetiology.

The evaluation of flushing in the maintenance phase (percentage of days with moderate or greater GFSS during weeks 2 through 8) was of particular interest as scant data assessing chronic ER niacin-induced flushing exist because of poor persistence with niacin therapy. There appears to be an inconsistency between the relatively low discontinuation rates reported in clinical trials of ER niacin and the poor persistence with ER niacin observed in clinical practice (7–9). The present study demonstrated that although the flushing signal was modestly attenuated in the second week, the frequency of flushing remained constant through the remaining weeks; flushing severity and bother with ER niacin continued to be greater than that with placebo for the duration of the study. Overall, 40% of patients taking ER niacin vs. 8% of patients taking placebo had more than 1 day per week with moderate or greater (GFSS ≥ 4) flushing. These results are generally consistent with the previously reported experience with niacin therapy in patients with dyslipidaemia.

Overall, 8% of patients discontinued because of flushing, none of whom came from the placebo group. Most discontinuations occurred within the first week of the study and there was a clear relationship between flushing severity or bother and the decision to discontinue therapy. Future studies will be required to link the magnitude and persistence of ER niacin-induced flushing with discontinuation behaviour.

Compliance with study drug (92%) and e-diary completion (87%) were high and not typical of real-world experience with niacin therapy; this study was designed and monitored to maintain optimal treatment compliance. Overall, ER niacin was well tolerated (independent of flushing), with no serious, drug-related AEs observed in this 8-week study. As the duration of this study is short, the observed lack of tolerability issues with ER niacin is not evidence of its long-term safety. The observed safety profile in the present study is consistent with that described in the ER niacin label (6).

In summary, the FSQ and particularly, the GFSS is a useful patient-reported outcome measure to objectively assess ER niacin-induced flushing. The use of patient-reported measures to characterise ER niacin-induced flushing will assist in the development of novel strategies for improving tolerability and patient adherence to niacin, an agent proven to reduce cardiovascular risk.

## Funding

This study was supported by Merck & Co., Inc., Whitehouse Station, NJ, USA.
